# Late adolescent outcomes of different developmental trajectories of ADHD symptoms in a large longitudinal study

**DOI:** 10.1007/s00787-024-02516-5

**Published:** 2024-07-06

**Authors:** Lara Carter, Lydia Speyer, Arthur Caye, Luis Rohde, Aja Louise Murray

**Affiliations:** 1 Department of Psychology, University of Edinburgh, 7 George Square, Edinburgh, EH8 9JZ UK; 2 Department of Psychology, University of Cambridge, Cambridge, UK; 3 ADHD Outpatient Program and Developmental Psychiatry Program, Hospital de Clinicas de Porto Alegre, Federal University of Rio Grande do Sul, Porto Alegre, Brazil; 4 Medical Council UNIFAJ and UNIMAX, São Paulo, Brazil; 5 National Institute of Developmental Psychiatry and National Center for Innovation and Research in Mental Health, São Paulo, Brazil

**Keywords:** Attention-deficit/hyperactivity disorder, Trajectories, Onset, Remission, Persistence, Adolescent outcomes

## Abstract

There exists substantial heterogeneity in the developmental trajectories of ADHD symptoms, with distinctions often made between persistent versus remittent, and early- versus late-onset. However, how these trajectories relate to late adolescent functioning and whether, in particular, later onset trajectories mark a milder subtype remains unclear. Building on earlier work that has examined early life predictors of ADHD symptom trajectories up to age 14, we applied latent class growth analysis to data from the UK Millennium Cohort Study (*N* = 10,262) to evaluate whether developmental trajectories of ADHD symptoms up to age 17 (from age 3) were similar to those identified up to age 14 and associated with differing levels of impairment in peer victimisation, mental health, substance use, and delinquency outcomes at age 17. Our optimal model included five trajectory groups, labelled *unaffected (37.6%), mildly affected (34.8%), subclinical remitting (14.4%), adolescent onset (7.6%),* and *stable high (5.6%)*. Adolescent onset and stable high trajectories were similarly impaired across all outcomes, other than substance use. Subclinical remitting individuals were impaired on self-esteem and well-being compared to unaffected individuals. By the end of mid-adolescence, those with a later onset have similar impairments to those following an early onset/persistent trajectory. Residual impairment may remain for those on a remitting trajectory.

**Supplementary Information:**

The online version contains supplementary material available at 10.1007/s00787-024-02516-5.

## Introduction

Attention-deficit/hyperactivity disorder (ADHD) is characterised by inattention and/or hyperactivity-impulsivity levels that interfere with functioning. Symptoms have been associated with a range of impairments including higher levels of substance use, criminality, co-occurring mental health issues, and social difficulties (e.g., [9]. Research has highlighted considerable heterogeneity in the lifespan course of ADHD symptoms [6]. Accordingly, researchers have begun to organise this variation into developmental ‘subtypes’ of ADHD, such as ‘early-onset persisting’, ‘early-onset remitting’ and ‘late-onset’, reflecting the primary ways in which symptoms are assumed to present over time [6]. However, less is known about whether such groups can be differentiated on the basis of clinically meaningful outcomes, which may suggest a benefit of diagnostic specifiers for ‘developmental subtype’. For example, demonstrating that some ADHD symptom trajectories are associated with greater or different patterns of impairment compared to others (e.g., higher rates of criminality, co-occurring conditions etc.), would suggest the need for tailored intervention strategies that target the particular needs of each trajectory group.

Analysing outcomes of different trajectories is also important for establishing whether there are impairments (relative to those who never show elevated ADHD symptoms) that outlast clinically significant symptoms for those who remit, implying that this group may require continued support beyond symptom remission. Likewise, given the application of age of onset cut-offs for ADHD, it is important to establish whether those who do not meet age cut-offs may nevertheless show impairments and benefit from intervention, despite not showing a ‘classical’ ADHD symptom trajectory. The extent to which this trajectory predicts outcomes associated with ADHD can inform current debates about the clinical validity of a ‘late onset’ category [12].

ADHD symptom trajectories and their outcomes have been studied predominantly through the a-priori classifications. For example, studies have classified symptoms as persisting or remitting depending on whether symptoms are clinically significant at both an early and later age (persisting) or at just an earlier age (remitting) and have tended to find poorer outcomes for symptom-persisting compared to symptom-remitting individuals (e.g., [2]. Studies have also compared early- versus late-onset ADHD based on whether symptoms first appear after versus before the age of onset in diagnostic criteria for ADHD (i.e., after age 12, previously 7). These studies have yielded somewhat mixed findings, however, most have found comparable levels of co-occurring mental health issues, delinquency, social difficulties, and tobacco, alcohol, and illicit drug use/misuse among early and late-onset subtypes (e.g., [1, 7, 10, 15]. However, these previous studies have not modelled the full variation that exists in ADHD symptom trajectories. For example, defining late-onset as age 12 or above (as is encoded in clinical diagnostic criteria) may be considered arbitrary because the emergence of clinically relevant symptoms can occur across a wide range of ages, possibly up to and including adulthood (e.g., [3]. Similarly, rather than there being a single point in time at which symptoms remit/appear, evidence points to continual fluctuations in symptoms over time for many [39].

To better reflect individual and developmental variation and thus better detect differences in outcomes, longitudinal studies drawing on data-driven techniques such as latent class growth analysis or growth mixture modelling can be employed. Such methods model linear and non-linear changes in symptoms over a developmental period to identify trajectory groups that optimally reflect patterns of symptom variation in a particular sample (e.g., [16, 18, 19, 21–27, 31, 32, 36, 41]. Whilst varying in terms of their samples, measurement methods, and developmental periods covered, certain commonalities have surfaced across the findings of such studies. Using these approaches, trajectories that could be mapped approximately to the early-onset persisting, early-onset remitting, and late-onset groups that are typically specified in studies using a-priori classification often emerge, however, with a more detailed picture of how symptoms develop over time.

ADHD symptom trajectories emerging from trajectory analyses can also be compared with respect to various outcome variables. Sasser et al. [36] used trajectory analysis with parent-reported ADHD symptom data across ages 8–18 and found that three trajectory groups emerged, labelled ‘low’ (consistently low symptom levels across time), ‘declining’ (symptoms that remitted over time) and ‘high’ (symptoms that persisted over time). Those in the high trajectory group had elevated rates of parent-reported antisocial behaviour and school dropout, but similar levels of unemployment and juvenile arrests compared to the declining group. Tandon et al. [41] also identified ‘low’, ‘remitting’ and ‘high’ trajectory groups when analysing ADHD symptoms across ages 9–21. On a range of psychiatric disorders (including major depressive and oppositional defiant disorder), the high group was found to have the most co-occurring issues, followed by the declining, and then the low group. However, rates of other disorders such as generalised anxiety disorder, alcohol and cannabis use disorder did not differ between any of the three groups. Murray et al. [18] found that their ‘late-onset’ group (characterised by rising symptoms across ages 7–15) and ‘persistent’ group (persistently high symptoms across ages 7–15) were similar to each other across most outcomes including comparably high rates of delinquency, internalising problems, violent ideations, and cigarette smoking in comparison to ‘unaffected’ individuals. However, consistent with some of the aforementioned a-priori studies, they also noted some poorer outcomes for early-onset individuals such as higher levels of reactive aggression compared to late-onset individuals, thus leading authors to conclude that late-onset may represent a milder, though still impaired, subtype of ADHD. This study provided some initial evidence on how ADHD symptom trajectories link to outcomes but the sample was considerably smaller than some other datasets that have relevant symptom trajectory data and though community-ascertained, was not nationally representative.

Trajectory analysis studies to date have thus started to suggest the possibility of differential impairments between groups with different developmental trajectories of ADHD symptoms. However, with such studies remaining relatively scarce and with the importance of such work for informing diagnostic and treatment procedures, further work is necessary to ensure the replicability of these preliminary findings. As such, in a large UK-representative sample, we sought to investigate different developmental trajectories of ADHD. In this, we build on a previous trajectory analysis study examining trajectories in the same sample [21]. However, in that study trajectories were only estimated up to the age of 14 based on data availability. As adolescence is a time of rapid and marked change, with specific implications for ADHD-related traits such as sensation-seeking and self-regulation [22–27, 37], it is important to build on these earlier analyses to examine how trajectories extend up to the end of middle adolescence. For example, it is unclear if ‘adolescent-onset’ trajectories might represent temporary versus sustained increases in symptoms, whether remitting trajectories tend to show an accelerating, decelerating, or stabilising trajectory towards the end of middle adolescence,or whether there may be entirely new trajectory classes emerging (e.g., with an onset later in adolescence) when a more extended developmental period is considered. Further, whilst these earlier analyses sought to validate and provide a broader characterisation of trajectory-based distinctions by examining early-life predictors and possible etiological of correlates of trajectory analysis membership, they did not examine *outcomes* of following particular trajectories. This is arguably of greater immediate clinical relevance than identifying early-life predictors of class membership as they can inform the provision of tailored preventive interventions and support to mitigate anticipated challenges.

In this study we, therefore, extend earlier analyses to examine developmental trajectories, now using data from ages 3–17 and also examine the links between these trajectories and outcomes at age 17. We examine whether age 3–17 trajectories are associated with differing levels of impairment on multiple outcome variables that have previously been associated with ADHD symptoms, assessed when participants were aged 17. Outcomes included substance use, dimensions of mental health (self-esteem, psychological distress, and well-being), peer victimisation, and delinquency. Though specific hypotheses are difficult to define prior to selecting a trajectory model, based on trends from previous research, we hypothesised that the pattern across outcome variables would be: (1) those with persistently high ADHD symptom levels will have the most impairment compared to relevant other groups whilst unaffected individuals will have the least impairment, (2) those with a late-onset of symptoms will have fewer impairments than those with an early-onset persistent but more impairments than unaffected individuals, and (3) those whose symptoms remit will have fewer impairments than those whose symptoms persist but more impairments than unaffected individuals.

### Methods

#### Participants

Participants (*N* = 10,262) were from the Millennium Cohort Study (MCS; [8] who provided data across sweeps 2–7 of the study (average age at each sweep was 3, 5, 7, 11, 14 and 17 years, with information about the age distributions at each sweep provided in Supplementary Materials Table S1). MCS has been tracking the development, family, and wider social lives of individuals born in the United Kingdom (UK) between 2000 and 2002. Participants were sampled using a stratified, clustered random sampling design in which individuals were clustered geographically and disproportionately stratified so as to over-sample residents of the three smaller countries of the UK (Scotland, Wales and Northern Ireland), disadvantaged areas and ethnic minorities. As such, sampling weights were used in all analyses to adjust for the effects of attrition and non-random sampling, thus ensuring results were UK-representative. For further details, the MCS is fully documented and freely accessible at: https://ukdataservice.ac.uk/. Written/verbal informed consent was obtained from all parents/participants where required. Ethical approval for the current secondary data analysis was granted by the University of Edinburgh School of Philosophy, Psychology and Language Sciences Ethics Committee.

### Measures

#### ADHD symptoms

ADHD symptoms were measured using the Strengths and Difficulties Questionnaire (SDQ; [11]. The SDQ is one of the most widely used and well-validated behavioural screening instruments for children and adolescents (Kersten et al., 2016). It has shown good psychometric properties in the current sample, including a high degree of gender and developmental invariance [25]. The hyperactivity/inattention subscale has shown high correlations with ADHD diagnosis [32]. It includes five items asking parents about their child’s behaviour during the last six months with reference to the following behaviours: ‘restless, overactive, cannot stay still for long’,‘constantly fidgeting or squirming’,‘easily distracted, concentration wanders’,‘thinks things out before acting’; and ‘sees tasks through to the end, good attention span’. At age 3, the item ‘thinks things out before acting’ was replaced with ‘can stop and think things out before acting’ to improve its age-appropriateness. Responses were recorded on a 3-point scale including *not true* (0), *somewhat true* (1), and *certainly true* (2). Positively worded items were reverse-coded, and responses were summed to produce an overall hyperactive/inattentive score with higher scores indicating greater hyperactivity/inattentiveness (possible range = 0–10). In a similar sample to the current study, Riglin et al. [32] found an optimal cut-off for identifying clinically significant symptoms (assessed via a *DSM-IV* diagnostic interview) to be a score of 7 or more on the SDQ subscale (specificity = 90%, sensitivity = 86%, area under the curve = 0.88), with 6 representing a borderline score.

### Adolescent outcome measures

All outcomes were assessed when participants were aged 17 via online self-report questionnaires and face-to-face interview. Measures are described below with further details in Supplemental Materials. Outcome variables were selected based on the availability of measures of concepts that have been associated with ADHD symptoms in previous research.

#### Peer victimisation

Peer victimisation was assessed as the number of victimisations the participant had experienced in the last 12 months. Six items (*α* = 0.70) measured whether participants had experienced a range of verbal, physical, emotional, and online abusive behaviours (e.g., ‘Has anyone called you names?’, ‘Has anyone been physically violent towards you?’). Participants responded ‘no’ (0) or ‘yes’ (1) to each item and responses were summed to create a total victimisation score for each participant, with higher scores reflecting a greater number of victimisation behaviours experienced (range = 0–6).

#### Substance use

To assess alcohol consumption, participants were asked ‘How many times have you had an alcoholic drink in the last 12 months?’ with responses recorded on a 7-point scale (*Never* = 0, *1–2 times* = 1, *3–5 times* = 2, *6–9 times* = 3, *10–19 times* = 4, *20–39 times* = 5, *40 or more times* = 6). One item assessed participants’ cannabis use: ‘In the past year how many times have you taken cannabis?’ with responses recorded on a 5-point scale (*Not taken in the last year* = 0, *1–2 times* = 1, *3–4 times* = 2, *5–10 times* = 3, *More than 10 times* = 4).

#### Mental health

Anxiety and depressive symptoms over the last 30 days were assessed via the 6-item Kessler Psychological Distress Scale [13], *α* = 0.86). Responses were on a 5-point scale ranging from *none of the time* (1) to *all of the time* (5) and summed to create a total composite score (range = 6–30). Mental wellbeing over the past two weeks was measured via the 7-item Short Warwick-Edinburgh Mental Wellbeing Scale [40], *α* = 0.83). Responses were on a 5-point scale from *none of the time* (1) to *all of the time* (5). Positively worded items were reverse-coded, and responses summed to create a total composite score (range = 7–35). Global self-esteem was measured using five items from the Rosenberg Self-esteem Scale [33], *α* = 0.91). Responses were on a 4-point scale from *strongly disagree* (1) to *strongly agree* (4). Positively worded items were reverse-coded, and responses summed to create a total composite score (range = 5–20). Higher scores on each of the three scales indicated greater impairment.

#### Delinquency

Delinquency was measured via nine items (*α* = 0.65) assessing whether participants had engaged in various delinquent behaviours during the past 12 months including theft, vandalism, breaking and entering, arson and online hacking (e.g., ‘Have you taken something from a shop without paying for it?’, ‘Have you deliberately set fire to something that you shouldn’t have?’). Participants responded ‘no’ (0) or ‘yes’ (1) to each item and responses were summed to create a total delinquent score for each participant, with higher scores indicating greater delinquency (range = 0–9). This ‘variety index’ score method of measuring delinquency is recommended (as opposed to summing the frequency of individual behaviours) because it avoids scores being disproportionately influenced by non-serious but frequent delinquent acts.

### Model selection

Latent class growth analysis models were fit with increasing numbers of classes until a stopping point was reached, defined by a non-significant Lo-Mendell-Rubin (LMR) adjusted test. Akaike’s information criterion (AIC), Bayesian information criterion (BIC), and sample size adjusted BIC (saBIC) were used to help with model selection in cases where the adjusted LMR test yielded ambiguous results. It is known that no single class enumeration will consistently select the ‘correct’ number of classes and as such class enumeration indices are best used to inform the numbers of classes alongside substantive and pragmatic criteria such as the interpretability of classes [30, 43]. Moreover, when conceptualizing the latent class models as a means of providing a convenient but defensible discretization of an underlying continuous distribution, as we do in the current context, there is no ‘correct’ number of classes to detect, only an optimal number for summarising variation in a parsimonious manner [28].

Growth models with intercept, linear slope, and quadratic slope factors included were fit based on previous research suggesting that ADHD symptom trajectories tend to be curvilinear [18, 22]. Time was scaled by fixing the slope factor loadings proportional to the distance between waves with age 3 loadings fixed to 0 (baseline) and age 17 loadings fixed to 1. Factor variances and covariances were fixed to 0 within classes, implying that all trajectory variation is due to the underlying latent categorical variable. This operationalises the assumption of the latent classes as convenient summaries of an underlying continuous distribution rather than necessarily reflecting true typologies (see [29] for a discussion). This can be contrasted to a growth mixture modelling (GMM) approach which has been interpreted conceptualising groups in terms of subpopulations (see e.g., [29]. Given this conceptualisation, GMM thus allows variation around an average growth curve within each subpopulation. As a consequence of allowing this within-class variation, it typically models the same data using fewer sub-groups.

### Outcomes of ADHD symptom trajectories

Following the selection of an optimal latent class growth analysis model, age 17 outcomes were compared across classes, using the three-step method described in Asparouhov and Muthén [4] to correct for classification uncertainty. This method can be vulnerable to changes in the nature of the classes with the inclusion of outcomes in the model,however, this is checked and flagged by the analysis programme when it arises. If this occurred for a given outcome we used the BCH method discussed in Asparouhov and Muthén [4]. The BCH method involves fitting a multi-group model treating the class membership as known and weighting observations based on weights derived from their classification probabilities. Most likely class membership and classification probabilities are obtained from the latent class growth analysis model estimated in the first step. All outcomes were treated as continuous as they had a minimum of five response options. All analyses were conducted in Mplus 8.4 using (robust) pseudo-maximum likelihood estimation that adjusts for the complex sampling design of MCS [27]. Missing data were dealt with using attrition weights provided by MCS. These up-weight respondents with a low probability of responding and down-weight those with a high probability to correct for non-random drop-out. This provides unbiased parameter estimates under a ‘missing at random’ (MAR) assumption in Rubin’s [34] terminology.

## Results

**Fig. 1:**
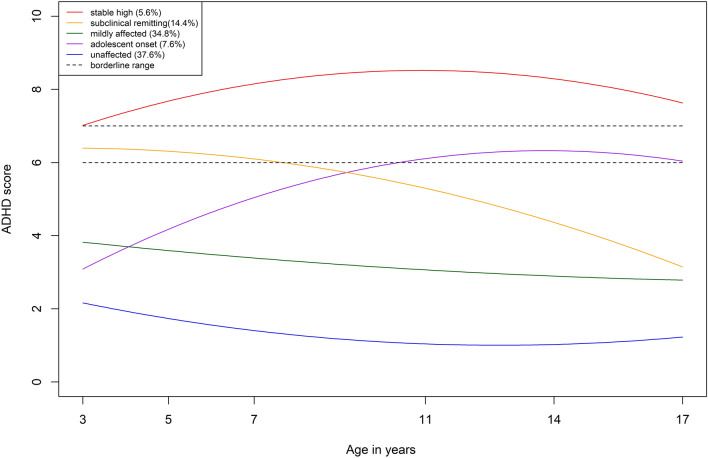
ADHD symptom trajectory groups. Figure shows the estimate trajectories for each group based on the intercept and slope factor means from the optimal model

Descriptive statistics can be found in Table S1 of Supplementary Materials. Model fits for the latent class growth analysis models with between 1 and 8 classes are provided in Table S2 of Supplementary Materials. Full model outputs are provided at: https://osf.io/ghvt5/. The LMR test pointed to either a 3- or 5-class model as optimal; however, the 5-class model had substantially lower information theoretic criteria values and it made substantively important class distinctions that were blurred in the 3-class model. The 5-class model is summarised in Table 1 and Fig. 1.

**Table 1:** Model parameters for the 5-class model

Class	Class label	Class size*	Intercept factor mean	Intercept factor mean SE	Linear slope factor mean	Linear slope factor mean SE	Quadratic slope factor mean	Quadratic slope factor mean SE
1	Adolescent onset	7.6% (n = 778)	3.088	0.230	8.398	0.867	−5.446	0.772
2	Mildly affected	34.8% (n = 3579)	3.824	0.109	− 1.711	0.280	0.672	0.290
3	Unaffected	37.6% (n = 3857)	2.163	0.051	− 3.337	0.182	2.403	0.167
4	Stable high	5.6% (n = 572)	7.018	0.180	5.316	0.776	−4.704	0.711
5	Subclinical remitting	14.4% (n = 1479)	6.395	0.142	− 0.154	0.642	−3.095	0.549

*SE* standard error

*Based on posterior probabilities

Trajectories tended to show curvilinear change and were differentiated in both pattern and severity (and they did not merely show a ‘cat’s cradle’ pattern that may be characteristic of LGCA solutions in developmental science [38]. An ‘unaffected’ class (37.6%) was characterised by consistently low levels of symptoms throughout the developmental period studied. A ‘mildly affected’ class (34.8%) was characterised by consistently slightly elevated levels but that remained far from the borderline threshold throughout the developmental period studied. A ‘subclinical remitting’ class (14.4%) was characterised by symptom levels that began in the borderline range but which declined from there. Beyond age 7, the symptom levels of this group were no longer in the borderline range and by age 17 they had reached similar levels to the ‘mildly affected’ class. An ‘adolescent onset’ class (7.6%) was characterised by initially low symptom levels that increased gradually over childhood to reach borderline levels in adolescence. Finally, a ‘stable high’ class (5.6%) was characterised by symptom levels that were already in the clinical range by age 3 and remained in that range thereafter. They also showed a slight peak around late childhood/adolescence.

### Outcomes of trajectory class membership

The comparisons of outcomes across class groups are provided in Tables 2 and 3. We also focus on sets specific of contrasts most relevant for the specific hypotheses outlined above in light of the trajectory groups that were present in the selected longitudinal latent growth analysis: unaffected vs adolescent onset (relevant for hypothesis 2), unaffected vs stable high (relevant for hypothesis 1), adolescent onset vs stable high (relevant for hypotheses 1 and 2), unaffected vs subclinical remitting (relevant for hypothesis 3), and stable high vs sub-clinical remitting (relevant for hypotheses 1 and 3).

**Table 2:** Relations between ADHD trajectory class membership and age 17 outcomes

Outcome	Class mean (SE)				
	Unaffected	Mildly affected	Subclinical remitting	Adolescent onset	Stable high
Peer victimisation	1.074 (0.042)	1.321 (0.076)	1.285 (0.135)	1.645 (0.225)	1.507 (0.199)
Psychological distress	12.808 (0.150)	13.395 (0.353)	13.152 (0.330)	15.223 (0.789)	13.971 (0.519)
Poorer well-being	16.737 (0.140)	17.571 (0.480)	17.542 (0.387)	19.211 (0.812)	18.440 (0.465)
Lower self-esteem	9.689 (0.089)	9.947 (0.216)	10.295 (0.175)	10.612 (0.412)	10.339 (0.303)
Delinquency*	0.138 (0.014)	0.206 (0.027)	0.193 (0.051)	0.392 (0.071)	0.276 (0.058)
Alcohol use	2.982 (0.065)	2.642 (0.114)	2.300 (0.120)	2.143 (0.183)	2.284 (0.148)
Cannabis use	0.591 (0.044)	0.681 (0.053)	0.678 (0.125)	0.788 (0.143)	1.0730 (0.208)

*Using the BCH method due to class shifts using the three-step method

**Table 3:** Key trajectory class contrasts

Outcome	Unaffected vs subclinical remitting			Unaffected vs adolescent onset			Unaffected vs stable high			Stable high vs subclinical remitting			Stable high vs adolescent onset		
	${\chi }^{2}$	*p*	SMD	${\chi }^{2}$	*p*	SMD	${\chi }^{2}$	*p*	SMD	${\chi }^{2}$	*p*	SMD	${\chi }^{2}$	*p*	SMD
Peer victimisation	2.223	0.136	− 0.185	6.214	**0.013**	0.381	4.718	**0.030**	0.295	0.710	0.399	0.104	0.180	0.671	− 0.065
Psychological distress	0.942	0.332	− 0.138	8.775	**0.003**	0.482	4.874	**0.027**	0.039	1.542	0.214	0.128	1.469	0.225	− 0.205
Well− being	4.099	**0.043**	− 0.253	9.190	**0.002**	0.579	12.258	< 0.001*	0.414	1.801	0.180	0.167	0.594	0.441	− 0.170
Self− esteem	9.003	**0.003**	− 0.247	4.844	**0.028**	0.366	4.146	**0.042**	0.232	0.014	0.904	0.001	0.255	0.613	− 0.127
Delinquency	1.033	0.309	− 0.087	12.644	< 0.001*	0.339	5.099	**0.024**	0.205	1.037	0.308	0.101	1.461	0.227	− 0.124
Alcohol use	27.733	< 0.001*	0.354	18.214	< 0.001*	− 0.431	18.507	< 0.001*	− 0.392	0.007	0.935	− 0.028	0.322	0.570	0.056
Cannabis use	0.455	0.500	− 0.089	1.921	0.166	0.175	5.117	**0.024**	0.327	2.063	0.151	0.229	1.040	0.308	0.149

*SMD* standardised mean difference, standardised with respect to the pooled standard deviation of the outcome across the two groups and calculated using model constraints within the BCH manual method, as described in [4]. Significant *p*-values (< 0.05) indicated in boldface. A Bonferroni corrected threshold can also be defined for the family of tests (35 tests, 5 comparisons for each of 7 outcomes) as *p* < 0.0014. Effects that survive this correction are marked with a*

At odds with our first and second hypothesis, there were no significant differences between the stable high and adolescent onset groups. However, consistent with our first and second hypothesis, those in the adolescent onset class had consistently poorer outcomes than those in the unaffected class. There were only two exceptions: alcohol use, where the adolescent onset (as well as the high stable) group used significantly less than the unaffected group and cannabis use where the adolescent onset class did not differ significantly from the unaffected group, the latter likely reflecting that there was low frequency of usage in the sample overall. Only partially supporting our third hypothesis, there were inconsistent differences between the unaffected and subclinical remitting group: on well-being, self-esteem, and alcohol use but in the latter case it was the unaffected group that were the greater users. Finally, there were no significant differences between the stable high and sub-clinical remitting group, at odds with our third hypothesis. When applying a Bonferroni correction there were five significant comparisons remaining that might be considered particularly robust: Alcohol use in the unaffected vs remitting category; delinquency and alcohol use in the unaffected vs adolescent onset category; and well-being and alcohol use in the unaffected vs stable high category.

Taken together, the hypothesis that an early onset (‘stable high’) group would be the consistently most impaired was not supported based on the fact that there were no significant differences between the high stable and adolescent (‘late’) onset group. This result was also inconsistent with the hypothesis that a late onset group would show intermediate impairment between an unaffected and early onset group: the adolescent onset group was significantly different from the unaffected group on the majority of outcomes and not significantly different from the stable high group on any. The majority of contrasts were in line with the hypothesis that the unaffected class would evidence the least impairment. Finally, the hypothesis that those showing remitting symptoms would show intermediate impairments between the ‘stable high’ and unaffected group was not supported as this group showed some significant differences as compared to the ‘unaffected’ group but none compared to the ‘high stable’ group.

## Discussion

Using a large UK-representative sample, we investigated whether developmental trajectories of ADHD symptoms could be differentiated with respect to various age-17 outcomes. Whilst we did not know a priori what trajectory groups would emerge, we hypothesised that any early-onset persistent category that emerged would show the highest level of impairment and that any later-onset category or remitting categories would both show impairment intermediate between unaffected and early-onset/persistent at age 17. In fact, groups corresponding to these trajectories did emerge: in a latent class growth analysis, the model judged to best capture heterogeneity in ADHD symptom developmental trajectories had five classes, labelled: adolescent onset (7.6%), mildly affected (34.8%), unaffected (37.6%), stable high (5.6%; the only group crossing a clinical threshold), and subclinical remitting (14.4%). These trajectories were mostly similar to/or looked like developmental extensions of the trajectories estimated in Murray et al. [22], which only estimated trajectories up to age 14. The main difference was that a six- class solution was optimal in that study, which differentiated two ‘declining symptoms’ categories depending on whether they began in the borderline or subclinical range.

Results from our pairwise comparisons were, however, only partially consistent with our hypotheses. Specifically, both the adolescent onset and stable high groups had worse scores than the unaffected group on almost all outcomes studied (except substance use) but did not differ significantly from each other. As such, the adolescent onset was similar to the stable high group rather than showing intermediate levels of impairment. Further, the subclinical remitting group showed worse self-esteem and well-being than the unaffected group but otherwise did not differ significantly from this group nor the stable high group. Taken together, our findings support the recognition and potential need for intervention in later onset trajectories of ADHD symptoms due to the associated impairment. Further, there is some evidence for poorer outcomes even for those showing a declining symptoms trajectory.

The trajectory groups that emerged from the present study are largely consistent with previous studies that have tended to find that for those affected by ADHD symptoms, some will show early emerging and persistent symptoms, some will show remitting symptoms, and others still will not show an escalation of symptoms until later in development (e.g., [18, 19, 31]. The trajectories were also similar to a previous analysis that modelled the trajectories of ADHD symptoms in the sample up to age 14 [22]. The current analyses, which extended the age range up to age 17, suggest that adolescent-emerging symptoms do not merely show a transient increase but can continue to show high symptom levels up to the end of middle adolescence.

To provide further illumination on the nature of this later onset category, we compared the adolescent onset trajectory to the unaffected and stable high trajectory groups and found that although an adolescent onset subtype showed worse age-17 scores on almost all outcomes studied, they did not differ significantly from those with an early onset and persistence (the ‘stable high’ group). While the previous literature is not entirely consistent on this (e.g., Karam et al., 2009; [31], our findings add to other emerging evidence that suggests that while those with a later onset may show fewer problems early in life (e.g., [22]), by the time they reach adolescence, they mostly show similar impairments to those with an early onset (e.g., [1, 2, 18, 19]. Taken together, this evidence supports the recognition of and the potential need for provision of interventions for ADHD symptoms irrespective of whether they first emerge in early childhood or later in development. Indeed, frontline professionals should remain attuned to later emerging symptoms, given persisting perceptions of ADHD symptoms as only early-emerging. Further research is necessary to illuminate how the trajectories of the adolescent onset group develop into adulthood (i.e., whether this group reflects a transient increase over adolescence), and to establish the extent to which this group is etiologically distinct from the early onset group despite the similarity of their impairments (e.g., whether the elevation of inattention and hyperactivity/impulsivity symptoms better reflects the onset of underlying internalising problems).

A second set of comparisons addressed the impact of remitting symptom trajectories, by comparison of our ‘subclinical remitting’ group to the ‘unaffected’ and ‘stable high’ groups. Only a small number of differences were found between the remitting and unaffected group, with the former showing reduced wellbeing and self-esteem, and no differences with the stable high group. Indeed, consistent with previous studies [1, 2, 6, 41], a qualitative comparison suggested that the remitting group was generally intermediate in scores between the groups affected by symptoms at age 17 and those never affected by symptoms. It suggests that while symptoms may decline over development, it cannot be assumed that there will be no residual effects. Indeed, early psychosocial issues associated with ADHD symptoms such as academic failures and peer rejection could impact the development of self-concept and have a lasting impact on factors such as self-esteem and well-being. Further, it is common for ADHD symptoms to persist at a still-elevated level even among those on a declining symptom trajectory and in the current sample, those on the remitting trajectory continued to show symptoms that were of a higher level than those in the unaffected and mildly affected categories. Taken together, these findings underline the importance of continued support for youth whose symptoms no longer exceed borderline or clinical thresholds, especially in relation to well-being and self-esteem.

Finally, one perhaps counterintuitive finding was that groups affected by ADHD symptoms reported consuming less alcohol in the past 12 months compared to unaffected individuals. Adolescence is a sensitive period for exposure to substances and indeed, there exists a well-documented link between ADHD and increased substance use (e.g., [14]. One possible explanation is that social impairments typically seen in ADHD may shield individuals from exposure to the social settings in which adolescent alcohol consumption typically occurs, thus *decreasing* alcohol use at this developmental stage [17].

### Limitations and future directions

Whilst our measure of ADHD symptoms is well-validated (Kersten et al., 2016), and includes items for inattentive and hyperactive-impulsive symptoms, it is only 5 items long and does not include separate validated subscales for these dimensions. This is a limitation as previous research suggests dissociable developmental trajectories for these symptoms, with distinct effects on later adolescent functioning [18, 19, 31]. There is also no ADHD diagnostic assessment available in MCS (only a single parent-report item), meaning that it is not possible to be certain which young people meet diagnostic criteria for ADHD. Further, only parent-reported data was available to assess ADHD symptoms across the entire developmental period of age 3 to 17,however, an important criterion for an ADHD diagnosis is that symptoms must occur trans-situationally (APA, 2013). Finally, although this study was based on a strong longitudinal design, we did not analyse causal relationships between ADHD symptom trajectories and outcomes, only associations. Based on the fact that there is currently insufficient field knowledge of the underlying causal structures relating ADHD symptom trajectories, outcomes, and their confounders it was not judged feasible to identify and adjust for a sufficient set of covariates in the study to support causal inference [42]. Compounding this is the fact that the dataset was not designed specifically to illuminate ADHD symptoms, or their trajectories, therefore, even if a sufficient set of covariates could be identified, they are unlikely to be available in full in the present dataset. As such, we elected to present only the basic associations between trajectory groups and outcomes, which can provide a foundation for future research addressing causal links. This was also consistent with our aim of establishing whether ADHD trajectories are *predictive* of outcomes, which could inform the provision of preventive interventions for anticipated outcomes irrespective of whether causality holds. However, future research could utilise a directed acyclic graph approach to develop hypotheses about the underlying causal structure that relates symptom trajectories to outcomes and that thus informs the identification, measurement of, and adjustment for a set of covariates (e.g., sex and gender, socioeconomic status, co-occurring conditions) to help support causal inference. For example, it would be expected based on past research that sex/gender could act as a confounder in the association between trajectory classes and outcomes, as there have been sex/gender differences identified in both in the past (e.g., [5,19,35]). There was also lack of information on intervention exposure in the present sample, therefore, we could not examine their role in trajectories. Future studies could thus also consider the role of interventions. Future studies could also examine a broader range of outcomes previously associated with ADHD symptoms and extend the analyses to later developmental stages. Further, future studies could compare and examine the joint trajectories of ADHD and commonly co-occurring difficulties, such as conduct problems, internalising problems, and peer problems [20,24,26]. This could help illuminate how ADHD co-develops with other issues, the extent to which findings of the current study are specific to ADHD trajectories, and establish how ADHD trajectories predict adolescent outcomes in the context of other symptom trajectories.

## Conclusions

UK adolescents from the general population with later onset ADHD symptom trajectories evidenced the same impairments in age 17 outcomes such as self-esteem, wellbeing, psychological distress, peer victimisation, and delinquency as those with an early onset trajectory with persistence. This supports the importance of recognising the impacts of ADHD even when symptoms do not have an early onset. Some residual impairments remain among those with a remitting trajectory, supporting continued additional support for these individuals, even after symptoms decline.

## Supplementary Information

Below is the link to the electronic supplementary material.

Supplementary file1 (DOCX 49 KB)

## Data Availability

Data used in the present study is available via the UK Data Service: UK Data Service.
