# Supplementary material for: Late adolescent outcomes of different developmental trajectories of ADHD symptoms in a large longitudinal study
Source: Eur Child Adolesc Psychiatry. 2024 Jul 6;34(2):709–19. doi: 10.1007/s00787-024-02516-5 (PMC11868152; doi:10.1007/s00787-024-02516-5)
Supplement: Supplementary file 1 — Supplementary file1 (DOCX 49 KB) [file 787_2024_2516_MOESM1_ESM.docx]

**Supplementary Materials**

Late adolescent outcomes of different developmental trajectories of ADHD symptoms in a large longitudinal study

European Child & Adolescent Psychiatry

Lara Carter^1^, Lydia Speyer^1, 2^, Arthur Caye^3^, Luis Rohde^3, 4, 5^, Aja Louise Murray^1*^

^1^Department of Psychology, University of Edinburgh, UK

^2^Department of Psychology, University of Cambridge, UK

^3^ADHD Outpatient Program & Developmental Psychiatry Program, Hospital de Clinicas de Porto Alegre, Federal University of Rio Grande do Sul, Brazil

^4^Medical Council UNIFAJ & UNIMAX, Brazil

^5^National Institute of Developmental Psychiatry & National Center for Innovation and Research in Mental Health, Brazil.

*Corresponding author at: Department of Psychology, University of Edinburgh, 7 George Square, Edinburgh, UK, EH8 9JZ; Email: aja.murray@ed.ac.uk

**Full Questionnaires for Participants**

1. **Peer victimisation**

| In the past 12 months has anyone done any of these things to you? |
| --- |
| Insulted you, called you names, threatened or shouted at you in a public place, at school, college or anywhere else? |
| Spread gossip about you, ignored you or you’ve experienced other emotional abuse? |
| Been physically violent towards you, e.g., pushed, shoved, hit, slapped or punched you? |
| Hit you with or used a weapon against you? |
| Stolen something from you e.g., a mobile phone, money etc.? |
| Harassed or bothered you via mobile phone or email? |
| *Response options for each: I do not wish to answer, Do not know, No, Yes* |

1. **Substance use**

| ***Alcohol consumption*** |
| --- |
| How many times have you had an alcoholic drink in the last 12 months? A drink is half a pint of lager, beer or cider, one alcopop, a small glass of wine, or a measure of spirits. If you have had more than one alcoholic drink at a time, count this as one time. |
| *Response options: I do not wish to answer, Do not know, Never, 1-2 times, 3-5 times, 6-9 times, 10-19 times, 20-39 times, 40 or more times* |
| ***Alcohol consumption (bingeing)***  How many times have you had five or more alcoholic drinks at a time in the last 12 months? A drink is half a pint of lager, beer or cider, one alcopop, a small glass of wine, or a measure of spirits. |
| *Response options: I do not wish to answer, Do not know, Never, 1-2 times, 3-5 times, 6-9 times, 10 or more times* |

| ***Cannabis use*** |
| --- |
| In the past year how many times have you taken cannabis? (also called Marijuana, Dope, Pot, Blow, Hash, Skunk, Puff, Grass, Draw, Ganja, Spliff, Smoke, Weed) |
| *Response options: I do not wish to answer, Do not know, Not taken in last year, Once or twice, Three or four times, Five to ten times, More than ten times* |

1. **Mental health**

| ***Kessler Psychological Distress Scale*** |
| --- |
| During the last 30 days, about how often did you feel so depressed that nothing could cheer you up? |
| During the last 30 days, about how often did you feel hopeless? |
| During the last 30 days, about how often did you feel restless or fidgety? |
| During the last 30 days, about how often did you feel that everything was an effort? |
| During the last 30 days, about how often did you feel worthless? |
| During the last 30 days, about how often did you feel nervous? |
| *Response options for each question: I do not wish to answer, Do not know, None of the time, A little of the time, Some of the time, Most of the time, All of the time* |

| ***Short Warwick-Edinburgh Mental Wellbeing Scale*** |
| --- |
| Please select the answer that best describes your experience of each over the last two weeks. |
| I’ve been feeling optimistic about the future |
| I’ve been feeling useful |
| I’ve been feeling relaxed |
| I’ve been dealing with problems well |
| I’ve been thinking clearly |
| I’ve been feeling close to other people |
| I’ve been able to make up my own mind about things |
| *Response options for each: I do not wish to answer, Do not know, None of the time, Rarely, Some of the time, Often, All of the time* |

| ***Rosenberg Self-esteem Scale*** |
| --- |
| How much do you agree or disagree with the following statements about you? |
| On the whole, I am satisfied with myself |
| I feel I have a number of good qualities |
| I am able to do things as well as most other people |
| I am a person of value |
| I feel good about myself |
| *Response options for each: I do not wish to answer, Do not know, Strongly disagree, Disagree, Agree, Strongly agree* |

1. **Delinquency**

| In the last 12 months have you done any of the following things? |
| --- |
| Taken something from a shop without paying for it? |
| Written things or spray painted on a building, fence or train or anywhere else where you shouldn’t have? |
| Deliberately damaged something in a public place that didn’t belong to you, for example by burning, smashing or breaking things like cars, bus shelters and rubbish bins? |
| Gone into someone’s home without their permission because you wanted to steal or damage something? |
| Stolen a vehicle that didn’t belong to you? |
| Deliberately set fire to something that you shouldn’t have? |
| Used someone else’s credit/debit card or bank account details, to buy things, or obtain money, without the owner’s permission? |
| Accessed, or hacked into, someone else’s internet-enabled device (e.g. computer, tablet, mobile phone, games console), e-mail or social networking account without their permission? |
| Used the internet to send viruses, spyware or other harmful software/malware, to deliberately damage or infect other computers? |
| *Response options for each: I do not wish to answer, Do not know, No, Yes* |

**Table S1**

*Descriptive statistics*

|  | ***N*** | **Mean** | ***SD*** |
| --- | --- | --- | --- |
| ADHD age 3 | 14156 | 3.885 | 5.647 |
| ADHD age 5 | 14190 | 3.167 | 5.486 |
| ADHD age 7 | 12980 | 3.274 | 6.321 |
| ADHD age 11 | 10939 | 2.984 | 6.097 |
| ADHD age 14 | 12237 | 3.146 | 6.567 |
| ADHD age 17 | 8924 | 2.651 | 5.530 |
| Peer victimisation | 10039 | 1.207 | 1.423 |
| Alcohol use | 10058 | 2.573 | 1.996 |
| Cannabis use | 10060 | 0.603 | 1.222 |
| Psychological distress | 10060 | 13.286 | 4.930 |
| Well-being | 10036 | 17.433 | 4.795 |
| Self-esteem | 10052 | 9.981 | 3.194 |
| Delinquency | 9943 | 0.190 | 0.658 |
| **Age distributions of participants at each sweep*** | | | |
|  | **Minimum** | **Median** | **Maximum** |
| Sweep 2 | 2.67 years | 3.08 years | 4.58 years |
| Sweep 3 | 4.41 years | 5.24 years | 6.17 years |
| Sweep 4 | 6.33 years | 7.25 years | 8.17 years |
| Sweep 5 | 10.17 years | 11.17 years | 12.33 years |
| Sweep 6 | 13.08 years | 14.25 years | 15.25 years |
| Sweep 7 | 16.08 years | 17.17 years | 18.33 years |

*Note.* *Approximated given that birth and interview dates are available only to the nearest month.

**Table S2**

*Model fits for latent class growth analysis models*

| Number of classes | LMR | *p* | AIC | BIC | saBIC | Entropy |
| --- | --- | --- | --- | --- | --- | --- |
| **1** | - | - | 222508.021 | 222573.147 | 222544.546 | - |
| **2** | 15283.633 | <.001 | 206818.700 | 206912.771 | 206871.459 | .803 |
| **3** | **4753.717** | **<.001** | **201944.313** | **202067.328** | **202013.305** | **.785** |
| **4** | 1404.711 | .168 | 200509.580 | 200661.541 | 200594.805 | .735 |
| **5** | **1247.073** | **.030** | **199236.752** | **199417.657** | **199338.211** | **.749** |
| **6** | 582.375 | .602 | 198646.614 | 198856.464 | 198764.306 | .716 |
| **7** | 232.778 | .299 | 197860.165 | 198106.196 | 197998.149 | .660 |
| 8 | 275.187 | .672 | 197585.530 | 197860.505 | 197739.747 | .659 |

*Note.* Optimal model indicated in boldface. LMR= Lo-Mendell-Rubin value; AIC= Akaike Information Criterion; BIC= Bayesian Information Criterion; saBIC= sample size adjusted BIC.
